# Purging behaviors relate to impaired subjective sleep quality in female patients with anorexia nervosa: a prospective observational study

**DOI:** 10.1186/s13030-017-0107-7

**Published:** 2017-08-16

**Authors:** Tokusei Tanahashi, Keisuke Kawai, Keita Tatsushima, Chihiro Saeki, Kunie Wakabayashi, Naho Tamura, Tetsuya Ando, Toshio Ishikawa

**Affiliations:** 1 Department of Psychosomatic Medicine, Kohnodai Hospital, National Center for Global Health and Medicine, 1-7-1 Kohnodai, Ichikawa, Chiba 272-8516 Japan; 2 Department of Psychosomatic Medicine, Saiseikai Fukuoka General Hospital, 1-3-46 Tenjin, Chuo-ku, Fukuoka 810-0001 Japan; 3 Department of Psychosomatic Research, National Institute of Mental Health, National Center of Neurology and Psychiatry, 4-1-1 Ogawahigashi, Kodaira, Tokyo 187-8553 Japan

**Keywords:** Eating disorders, Binge-eating, Vomiting, Circadian rhythm, Sleep duration, Depression

## Abstract

**Background:**

We examined how purging behaviors relate to subjective sleep quality and sleep patterns and how symptoms of disordered eating behaviors relate to global sleep quality in female patients with anorexia nervosa (AN).

**Methods:**

Participants were new consecutive female inpatients with a primary diagnosis of AN admitted to the Department of Psychosomatic Medicine at Kohnodai Hospital between June 26 and December 25, 2015. We recorded patients’ habitual eating behaviors, laxative overuse, or uretic misuse, and administered the Japanese versions of the Pittsburgh Sleep Quality Index (PSQI-J) and Center for Epidemiologic Studies Depression Scale. Raw PSQI-J data were used to determine sleep patterns (sleep-onset time, wake-up time, and sleep duration). To examine how purging behaviors related to sleep quality, we compared variables between AN restricting type (ANr) and AN binge-eating/purging type (ANbp). Spearman’s rank correlation analysis was used to examine which potential factors influence global PSQI-J score.

**Results:**

Participants were 20 patients, of whom 12 had ANbp. Two ANr patients (25%) had global PSQI-J scores greater than 5, compared to 9 ANbp patients (75%; *P* < 0.05). Circadian rhythm disruption and abnormal sleep duration were significantly greater in ANbp patients than in ANr patients (*P* < 0.05). Global PSQI-J was significantly correlated with a diagnosis of ANbp (*ρ* = 0.525; *P* < 0.05), vomiting (*ρ* = 0.561; *P* < 0.05), and duration of illness (*ρ* = 0.536; *P* < 0.05).

**Conclusions:**

ANbp patients had worse global sleep quality and greater disrupted sleep than did ANr patients. This suggests that treatments focusing on sleep would be useful, especially for ANbp patients. Furthermore, vomiting and duration of illness should be considered essential factors related to impaired global sleep quality.

**Trial registration:**

Not applicable.

## Background

Regular sleep is essential for physical and mental health. On the physical side, sleep disturbances are associated with a greater risk of diabetes [1], while sleep apnea and cardiovascular disease have a bidirectional relationship [2]. On the mental side, psychiatric disorders (primarily mood or anxiety disorders) can lead to sleep disturbances, while, conversely, sleep complaints are included in the diagnostic criteria of many psychiatric disorders [3].

Sleep disturbances are also a common complication of eating disorders [4]. There are three primary eating disorders: anorexia nervosa (AN), bulimia nervosa (BN), and binge-eating disorder (BED) [5]. AN is characterized by extremely low body weight and body image distortion, accompanied by a fear of gaining weight. There are also two sub-types of AN: AN restricting type (ANr), wherein the individual restricts their food intake but does not exhibit purging behaviors (e.g., self-induced vomiting, misuse of laxatives or diuretics), and the AN binge-eating/purging type (ANbp), wherein the individual regularly engages in binge-eating or purging behaviors. The intense fear of gaining weight can cause patients with AN to experience extreme weight loss, without any relation to organic disease. The physical consequences of such weight loss can be life-threatening [6–9]. BN is characterized by recurrent binge-eating episodes accompanied by a sense of loss of control, as well as compensatory purging behaviors; patients with BN do not have low body weight, and their self-evaluations are extremely influenced by their body shape and weight. AN and BN can be regarded as similar pathological conditions, with patients showing transfer from one diagnosis to another [10, 11]. Finally, BED is characterized by eating excessive amounts of food recurrently, without purging behaviors or body weight loss. BED differs from AN or BN in that the criteria do not require extreme interest in body shape or weight [12]. We focused on AN in this study because patients with AN can exhibit purging behaviors or not.

Regarding the presence of sleep disturbance in these patients, a study of 549 college women revealed that 30% of participants with an eating disorder (AN, BN, or BED) based on the criteria of the Diagnostic and Statistical Manual of Mental Disorders, Fifth Edition (DSM-5) had significant insomnia; comparatively, only 5% of participants without an eating disorder had insomnia [13]. However, there remain some unanswered questions related to this topic, such as the factors that predispose individuals with eating disorders to sleep disturbances. There is some evidence that purging behavior is associated with an increased sleep disturbance: in our previous retrospective study of 1374 outpatients, patients with ANbp were found to experience greater sleep disturbances than were patients with ANr [14]. Moreover, a past study found that female outpatients with ANr and ANbp both had a relatively high prevalence of sleep disturbances, at 15.8% and 70.8%, respectively (it should be noted that this past study did not report a statistical comparison of these rates) [15]. Conversely, Lombardo et al. showed that the relationship between severity of insomnia and severity of eating disorder symptoms had only a weak direct link in female university students, but it had a strong indirect link via depression [16].

The current study was prospective, with the goal of understanding how purging behaviors relate to subjective sleep quality and sleep patterns and which disordered eating behaviors (e.g., binge-eating, vomiting, chewing, laxative overuse, or uretic misuse) are related to global sleep quality in female patients with AN. We also wanted to confirm the role of depression in these relationships. We hypothesized that patients with purging behaviors (i.e., ANbp) would have worse sleep quality and more disrupted sleep patterns and that depression and certain purging behaviors (i.e., vomiting) would be positively related to impaired global sleep quality.

## Methods

### Facility

All data were collected that the Department of Psychosomatic Medicine, Kohnodai Hospital, National Center for Global Health and Medicine, Chiba, Japan.

### Participants

Participants were consecutive female inpatients admitted this department for the first time between June 26 and December 25, 2015 (i.e., 6 months) and who had a primary diagnosis of AN according to DSM-5 criteria. This was determined by modifying the Japanese version of the structured clinical interview for the Diagnostic and Statistical Manual of Mental Disorders, Fourth Edition (DSM-IV), given that a DSM-5 version did not exist at the time of the study. Exclusion criteria were readmission to this department; being pregnant; having a serious physical illness; or having a serious mental illness, such as schizophrenia, bipolar disorder, substance use disorder (except for laxative or uretic use), dementia, or major depressive disorder with a high risk of suicide or self-injury.

### Measures

Data were collected via structured interviews and questionnaires.

#### General characteristics and disordered eating behaviors

Age, body mass index (BMI), duration of illness, menstruation, and use of sedative drugs were recorded. Patients were also asked about their habitual eating behaviors, wherein they selected one of the following four: binge-eating alone, vomiting, chewing (excessive chewing without swallowing), or no binge-eating/vomiting/chewing. Additionally, they were asked about laxative overuse or uretic misuse.

#### Questionnaires

The participants completed two questionnaires. First, they completed the Japanese version of the Pittsburgh Sleep Quality Index (PSQI-J) to assess sleep quality and disruption. This scale has established reliability and validity. It consists of 19 items organized in seven subscales. Each subscale has a score range of 0–3, and the scores for these subscales are then summed to arrive a global PSQI-J score with a total range of 0–21. Lower scores indicate better sleep. A cut-off of 5/6 was used for the global score in this study [17, 18]; any score greater than the cut-off was regarded as indicative of impaired sleep quality.

Furthermore, from the raw data of the PSQI-J, we recorded sleep-onset time, wake-up time, and sleep duration in order to examine the effects of sleep patterns. There are no universal criteria for abnormal sleep-onset and wake-up time, but considering past results for the Morningness-Eveningness Questionnaire (MEQ) [19] and analyses of Japanese social life [20], a sleep-onset time of before 21 h (9:00 pm) or after 25 h (i.e., 1:00 am; note that we put the limit of sleep-onset time at 25 h to be able to calculate a 24-h period; we also did not use the minute unit), and a wake-up time of before 5 h or after 9 h was regarded as having circadian rhythm disruption. Participants whose sleep duration was 10 h or more were considered long sleepers, and those with 5 h or less were considered short sleepers.

We also administered the Center for Epidemiologic Studies Depression Scale (CES-D) to assess depression. This scale also has established reliability and validity. It contains 20 items, each scored on a scale of 0–3; thus, the total score ranges from 0 to 60, with higher scores indicating greater depression. We employed a cutoff for risk of clinical depression of 15/16 [21, 22].

### Statistical analysis

First, we examined how purging behaviors related to sleep qualities by classifying participants into ANbp (i.e., report of vomiting, chewing, laxative overuse, or uretic misuse) and ANr. We then compared the PSQI-J between this and other variables.

Second, to determine the potential factors that influence global sleep quality (i.e., global PSQI-J score), we used Spearman’s rank correlation analysis. We could not perform a multiple regression analysis because of the small sample size.

## Results

### Participant characteristics

**Fig. 1:**
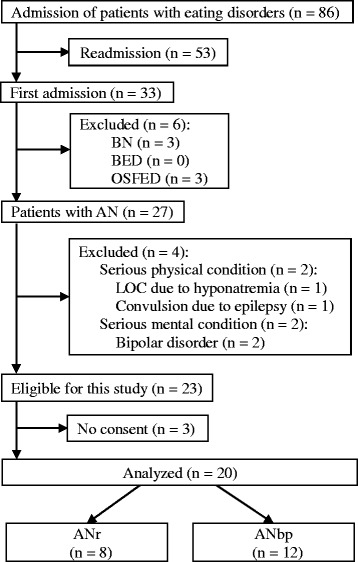
Participant flow chart. AN, anorexia nervosa; ANbp, anorexia nervosa binge-eating/purging type; ANr, anorexia nervosa restricting type; BED, binge-eating disorder; BN, bulimia nervosa; OSFED, other specified feeding or eating disorder; LOC, loss of consciousness

A flow chart of participation is shown in Fig. 1. Eighty-six patients with eating disorders were admitted during the research period, of whom 23 with AN were eligible. Three ANbp patients did not consent to this study. Thus, the data of 20 AN patients, all of whom were Japanese-speaking, were analyzed. The participant characteristics are shown in Table 1. Their mean age was 29 years (range = 15–58), and their mean BMI was 13.3 kg/m^2^ (range = 9.6–17.9). The mean duration of illness was 7.2 years (range = 0.7–37.0). The mean global PSQI-J score was 7.1 (range = 0–16); 11 (55%) of the patients had a global PSQI-J of greater than 5. The mean sleep-onset time was 23.9 h (range = 17.2–33.0). The mean wake-up time was 7.0 h (range = 3.0–16.0). Nine participants were considered to have circadian rhythm disruption, including five for the advanced sleep phase and four for the delayed sleep phase. The mean sleep duration was 6.8 h (range = 2.5–12.0). Six participants were considered to have abnormal sleep duration (2 long sleepers and 4 short sleepers). Twelve participants exhibited purging behaviors and were diagnosed with ANbp. Seven participants exhibited only vomiting; three exhibited only chewing; one exhibited both vomiting and laxative overuse; and one exhibited vomiting, laxative overuse, and uretic misuse.

**Table 1:** Participant characteristics and comparison of ANr and ANbp patients

	All (*n* = 20)	ANr (*n* = 8)	ANbp (*n* = 12)	P
Age at admission, years	28.6 ± 13.1	26.4 ± 17.1	30.1 ± 10.2	0.549
Body mass index, kg/m^2^	13.3 ± 2.2	12.6 ± 1.5	13.7 ± 2.5	0.256
Duration of illness, years	7.2 ± 8.9	2.6 ± 2.7	10.2 ± 10.4	0.031*
Current menstruation	3(15%)	0(0%)	3(25%)	0.193
Use of sedative drugs	5 (25%)	0(0%)	5(42%)	0.051
Habitual eating behaviors				
Binge-eating alone	0(0%)	0(0%)	0(0%)	
Vomiting	9 (45%)	0(0%)	9 (75%)	
Chewing	3 (15%)	0(0%)	3(25%)	
No binge-eating/vomiting/chewing	8(40%)	8(100%)	0(0%)	
Drug abuse				
Laxative overuse	2 (10%)	0(0%)	2(17%)	
Uretic misuse	1 (5%)	0(0%)	1(8%)	
CES-D, score (0–60)	28.3 ± 14.6	24.0 ± 16.5	31.1 ± 13.1	0.299
PSQI-J				
Global PSQI-J, score (0–21)	7.05 ± 4.21	4.63 ± 2.62	8.67 ± 4.38	0.031*
C1 subjective sleep quality, score (0–3)	1.50 ± 0.83	1.25 ± 0.71	1.67 ± 0.89	0.281
C2 sleep latency, score (0–3)	1.05 ± 1.15	0.38 ± 0.74	1.50 ± 1.17	0.027*
C3 sleep duration, score (0–3)	1.20 ± 1.06	1.00 ± 0.93	1.33 ± 1.16	0.504
C4 habitual sleep efficiency, score (0–3)	0.50 ± 1.00	0.00 ± 0.00	0.83 ± 1.19	0.034*
C5 sleep disturbances, score (0–3)	1.10 ± 0.64	1.00 ± 0.76	1.17 ± 0.58	0.583
C6 use of sleep medication, score (0–3)	0.60 ± 1.14	0.38 ± 1.06	0.75 ± 1.22	0.487
C7 daytime dysfunction, score (0–3)	1.10 ± 0.85	0.63 ± 0.74	1.42 ± 0.79	0.038*
Impaired sleep quality^a^	11(55%)	2(25%)	9(75%)	0.040†
Circadian rhythm disruption^b^	9(45%)	1(13%)	8(67%)	0.025†
Abnormal sleep duration^c^	6(30%)	0(0%)	6(50%)	0.024†

Figures are in n (%) or means ± SD. *P < 0.05 on t-test. †*P* < 0.05 on Fisher’s exact test. ^a^Global PSQI-J > 5 score. ^b^Sleep-onset time before 21 h or after 25 h, or wake-up time before 5 h or after 9 h. ^c^Sleep duration ≥10 or ≤5 h. *ANr* anorexia nervosa restricting type, *ANbp* anorexia nervosa binge-eating/purging type, *CES-D* Center for Epidemiologic Studies Depression, *PSQI-J* Japanese version of the Pittsburgh Sleep Quality Index

### Comparison between ANr and ANbp patients

Excluding purging behaviors, which were related to the diagnosis, we compared all variables between patients with ANr and ANbp (see Table 1). Current menstruation, use of sedative drugs, impaired sleep quality, circadian rhythm disruption, and abnormal sleep duration were compared using Fisher’s exact test, and the others using the t-test. We found that ANbp patients had a significantly longer duration of illness than did ANr patients (*P* < 0.05). There were no significant differences in age, BMI, current menstruation, use of sedative drugs, or CES-D between the groups. Concerning sleep quality, the mean global PSQI-J score of ANbp patients was significantly greater than was that of ANr patients (4.63 vs. 8.67, range = 2–10 vs. 0–16, ANr vs. ANbp, respectively; *P* < 0.05). Furthermore, two (25%) and nine (75%) of the patients with ANr and ANbp, respectively, had global PSQI-J scores of greater than 5 (*P* < 0.05). Regarding the subscales of the PSQI-J, the mean scores for sleep latency, habitual sleep efficiency, and daytime dysfunction of ANbp patients were significantly greater than were those of ANr patients (*P* < 0.05). The mean sleep-onset time of ANr and ANbp patients was 22.4 (range = 21.0–23.6) and 24.8 (range = 17.2–33.0) hrs, respectively, while the mean wake-up times were 5.6 (range = 3.0–7.0) and 7.9 (range = 3.5–16.0) hrs. One ANr patient had circadian rhythm disruption for the advanced sleep phase, while eight ANbp patients had such a disruption, four for the advanced sleep phase and four for the delayed sleep phase. A significantly greater number of ANbp patients had circadian rhythm disruption compared to ANr patients (*P* < 0.05). The mean sleep durations of ANr and ANbp patients were 7.0 (range = 6.0–8.5) and 6.7 (range = 2.5–12.0) hrs, respectively; abnormal sleep duration was significantly more prevalent in ANbp patients than in ANr patients (*P* < 0.05).

### Spearman’s rank correlation analysis of global sleep quality

Global PSQI-J was significantly correlated with a diagnosis of ANbp (*ρ* = 0.525; *P* < 0.05), vomiting (*ρ* = 0.561; *P* < 0.05), and duration of illness (*ρ* = 0.536; *P* < 0.05; see Table 2). Additionally, these three variables were significantly correlated with each other. More specifically, a diagnosis of ANbp was significantly correlated with vomiting (*ρ* = 0.739; *P* < 0.001) and duration of illness (*ρ* = 0.514; *P* < 0.05), and vomiting was correlated with duration of illness (*ρ* = 0.602; *P* < 0.01).

**Table 2:** Spearman’s rank correlation analysis of global sleep quality (global PSQI-J)

Variables	*ρ*	*ρ*
Diagnosis of ANbp	0.525	0.017*
Age	0.345	0.136
Body mass index	0.168	0.479
Duration of illness	0.536	0.015*
Current menstruation	0.281	0.230
Use of sedative drugs	0.332	0.152
CES-D	0.318	0.172
Vomiting	0.561	0.010*
Chewing	−0.061	0.798
Laxative overuse	0.407	0.075
Uretic misuse	0.280	0.232

*ANbp* anorexia nervosa binge-eating/purging type, *CES-D* Center for Epidemiologic Studies Depression, *PSQI-J* Japanese version of the Pittsburgh Sleep Quality Index, *ρ* Spearman’s correlation coefficient

*P < 0.05

## Discussion

In this study, we found that patients with ANbp had worse global sleep quality (including longer sleep latency, lower habitual sleep efficiency, and worse daytime function) and more disrupted circadian rhythm and abnormal sleep duration than did ANr patients, all of which supported our hypothesis. In the Spearman’s rank correlation analysis, diagnosis of ANbp, vomiting, and duration of illness were found to be significantly related to impaired global sleep quality in patients with AN. This supports our hypothesis that vomiting (a major purging behavior of ANbp) was a factor related to impaired global sleep quality; however, contrary to our hypothesis, depression was not related with impaired sleep quality.

### Subjective and objective sleep quality of AN

In this study, 75% of ANbp patients had impaired sleep quality, compared to the 25% of ANr patients. Furthermore, none of ANr patients used sedative drugs. This study analyzed subjective sleep, but it remains unclear whether the objective sleep quality of ANbp is also worse than that of ANr. One past study showed that self-reported or subjective scores of sleep quality improved after weight restoration in patients with ANr, but objective sleep quality (via polysomnography) remained the same before and after weight restoration [23]. In the Sleep Heart Health Study, the associations between subjective and objective sleep qualities are not always concordant [24]. To our knowledge, there have been no studies using polysomnography for ANbp patients, thus, the difference in objective sleep quality between ANr and ANbp should be examined in future studies.

### Comparison of subjective sleep quality between past research and this study

Kim et al. researched sleep disturbance among 400 female outpatients with eating disorders and reported the following prevalence rates of sleep disturbance by eating disorder (using the DSM-IV): ANr, 3 out of 19 (15.8%) participants, and ANbp, 46 out of 65 (70.8%) [15]. In our study, the prevalence of impaired sleep quality was higher than were those past rates, perhaps because the cut-off of PSQI-J might be different from the sleep disturbance criteria of Kim et al., who, notably, did not mention any criteria in their article. Nevertheless, the results of the current prospective study generally accorded with those of Kim et al. and our previous study, in which patients with ANbp had greater sleep disturbance than did patients with ANr) [14].

A previous cohort study showed that short sleep duration was associated with dieting, fasting, taking diet pills, and purging among high school students in the United States [25]. This finding is important because patients with the purging type of eating disorders might engage more often in such weight-control behaviors when compared to patients with the restricting type. Notably, however, we found that the difference in the PSQI-J subscale “C3 sleep duration” between patients with ANr and ANbp was not significant, perhaps because the subscale does not consider excessively long sleep (i.e., sleep duration for more than 7 h is scored as 0). Indeed, the difference in abnormal sleep duration between the patients with ANr and ANbp was significant.

### Night eating syndrome and circadian rhythm disruption

Recently, researchers have proposed criteria for night eating syndrome (NES) [26], a disorder characterized by evening hyperphagia or nocturnal ingestion with awareness of those eating episodes. A study showed that 9.4% of patients with AN and 40.6% of those with BN met the criteria for NES [27]. Although we did not directly determine whether participants had NES in our study, in light of the prevalence rates in this past study, we suspect that there were patients with NES in our sample. For patients with NES, the sleep quality would be poor because their circadian rhythms would be disrupted by their behaviors. Similarly, our study showed that 67% of patients with ANbp had circadian rhythm disruption. In contrast, about 90% of patients with ANr had normal circadian rhythms and normal sleep duration. Thus, there is a possible relationship between purging behaviors and abnormal sleep patterns. This is further supported by the fact that light therapy, a method of treating circadian rhythm sleep-wake disorders, is potentially efficacious for treating binge-eating and purging behavior [28].

### Factors related to impaired global sleep quality in AN patients

As mentioned above, the PSQI-J has a disadvantage in that it does not consider excessively long sleep duration or circadian rhythm disruption. Nevertheless, it is widely used to assess global sleep quality. We found that a diagnosis of ANbp, vomiting, and duration of illness were potential factors associated with the PSQI-J in patients with AN. However, importantly, these three factors were correlated with each other, which obscures their individual contributions. We could not perform a multiple regression analysis in this study because of the small sample size as well as the problem of multicollinearity. We hypothesize that vomiting, rather than a diagnosis of ANbp, is an essential factor related to sleep quality because vomiting is a major purging symptom of ANbp. Furthermore, duration of illness would be an essential associated factor because a long illness burden tends to lead to worsened sleep quality overall. Both of these points are consistent with our clinical experience that impaired sleep quality may be related with duration of illness and vomiting. However, ANbp patients had a significantly longer duration of illness than did ANr patients in the current study sample, which negates our ability to confirm these potential explanations. To clarify the issue, more detailed future research would be necessary.

### Depression and sleep quality

Lombardo et al. studied the relationship between poor sleep and severity of eating disorder symptoms in 562 female outpatients with eating disorders [29]. They evaluated eating disorder symptoms using the “body dissatisfaction,” “drive for thinness,” and “bulimia” subscales of the Eating Disorder Inventory-Two (EDI-2), and found that poor sleep at admission indirectly predicted these three eating disorder symptoms via the mediator of depression. Furthermore, 6-month persistence of poor sleep directly predicted drive for thinness and bulimia, and indirectly predicted body dissatisfaction and drive for thinness via depression. Another study reported that CES-D scores were related to global PSQI in a community sample [30]. In contrast, we evaluated the sleep quality of patients with AN at a single point in time and focused on purging behaviors. In our Spearman’s rank correlation analysis, depression was not correlated with sleep quality, despite past findings. We nevertheless suggest that depression would be a factor related to impaired sleep quality among patients with AN, which we intend to clarify in future study with a larger sample size.

### Electrolyte abnormalities and blood glucose variations might affect sleep quality

In this study, we did not evaluate electrolyte abnormalities or blood glucose level. Patients with purging behaviors often exhibit metabolic alkalosis and electrolyte abnormalities, such as hypokalemia and hyponatremia [31]. Although the effect of electrolyte abnormality on sleep has not been examined sufficiently yet, a relationship might nevertheless exist. For instance, in a single-blind crossover study, patients with type 2 diabetes and nocturnal hypoglycemia showed a decreased awakening response [32], while another study indicated that plasma ghrelin, insulin, and glucose level patterns after oral glucose administration differed between patients with ANr and those with ANbp [33]. In that study, the plasma glucose of patients with ANbp peaked at 60 min after oral glucose administration, after which it declined sharply. In contrast, the glucose of those with ANr increased gradually, peaking at 120 min and thereafter gradually decreasing. Considering these findings, sudden electrolyte abnormalities and blood glucose variations might occur during sleep if patients with purging behaviors engage in binge-eating or purging before bedtime. These should be explored in the future.

### Speculated mechanisms leading to impaired sleep

We speculate that many patients with habitual vomiting tend to engage in binge-eating at night, given the high rate of NES among individuals with eating disorders, which in turn might delay their sleep onset time (with an extreme delay, such as sleep onset being in the afternoon, being classified as advanced sleep onset). Furthermore, those patients might have sudden electrolyte abnormalities and blood glucose variations because of binge-eating/vomiting before bedtime, thereby resulting in poorer sleep quality (longer sleep latency, lower habitual sleep efficiency). It is also possible that their sleep duration would be shorter because they would stay up late eating but still wake up early in order to accomplish their daily routine or that their sleep duration would be too long because of illness-related interference with their social life. This would lead to worse daytime function. Nevertheless, all of this speculation must be verified in future research.

### Limitations

One limitation is the relatively small sample size. The sample size was small because we had intended to keep accurate data over one year, but ultimately decided to shorten the study period to six months because the main author had to transfer to another hospital. Nevertheless, despite the small sample size, we were able to present these important results.

The second limitation is our separation of habitual eating behaviors into binge-eating alone, vomiting, chewing, and no binge-eating/vomiting/chewing, despite the fact that most participants who had vomiting might have had binge-eating as well. Thus, we could not investigate how sleep quality was independently influenced by binge-eating. Furthermore, binge-eating/vomiting is a major set of disordered eating behaviors, and patients with self-induced vomiting to avoid weight gain might believe that they have eaten too much even if the actual volume of food consumed is low or normal. Additionally, we included “binge-eating alone” as a symptom, but no participant had it. Patients with BED would likely have had binge-eating alone, but these patients are less likely to be admitted to the study department because they tend to have less serious physical conditions. It would be necessary to investigate individuals with these symptoms in an outpatient department.

Finally, we defined circadian rhythm disruption using sleep-onset and wake-up time in this study. These criteria were meant to capture deviation from desired sleep phase, but they differ from the criteria for diagnosing circadian rhythm sleep-wake disorders. Moreover, our criteria ignored irregular sleep-wake rhythm. More detailed research that precisely assesses circadian rhythms would be needed.

## Conclusions

This is the first study to focus on how purging behaviors of participants with eating disorders relate to subjective sleep quality. AN patients with purging behaviors (i.e., ANbp) tended to have worse global sleep quality than did those without. Additionally, this is perhaps the first study to show that patients with purging behaviors are more likely to exhibit disrupted sleep patterns, including circadian rhythm disruption and abnormal sleep duration, than are those without. In the correlation analysis, a diagnosis of ANbp, vomiting, and duration of illness were potential factors related to global sleep quality. Of these three variables, we consider vomiting and duration of illness as essential factors related to impaired global sleep quality. Purging behaviors, especially vomiting, might be related to NES, electrolyte abnormalities, or blood glucose level, which in turn can lead to disruptions in subjective sleep quality and sleep patterns. Thus, the sleep quality and patterns of patients with eating disorders might be vulnerable to purging behaviors, suggesting that treatments focusing on sleep would be useful. Although depression was not correlated to global sleep quality in this study, it might be necessary to confirm this finding in a study with a larger sample.

## Abbreviations

AN: Anorexia nervosa
ANbp: Anorexia nervosa binge-eating/purging type
ANr: Anorexia nervosa restricting type
BED: Binge-eating disorder
BMI: Body mass index
BN: Bulimia nervosa
CES-D: Center for Epidemiologic Studies Depression Scale
DSM-5: Diagnostic and Statistical Manual of Mental Disorders, Fifth Edition
DSM-IV: Diagnostic and Statistical Manual of Mental Disorders, Fourth Edition
EDI-2: Eating Disorder Inventory-Two
LOC: Loss of consciousness
MEQ: Morningness-Eveningness Questionnaire
NES: Night eating syndrome
OSFED: Other specified feeding or eating disorder
PSQI-J: Japanese version of the Pittsburgh Sleep Quality Index
