# Correction to: Purging behaviors relate to impaired subjective sleep quality in female patients with anorexia nervosa: a prospective observational study

**DOI:** 10.1186/s13030-018-0124-1

**Published:** 2018-03-15

**Authors:** Tokusei Tanahashi, Keisuke Kawai, Keita Tatsushima, Chihiro Saeki, Kunie Wakabayashi, Naho Tamura, Tetsuya Ando, Toshio Ishikawa

**Affiliations:** 1 Department of Psychosomatic Medicine, Kohnodai Hospital, National Center for Global Health and Medicine, 1-7-1 Kohnodai, Ichikawa, Chiba, 272-8516 Japan; 2 Department of Psychosomatic Medicine, Saiseikai Fukuoka General Hospital, 1-3-46 Tenjin, Chuo-ku, Fukuoka, 810-0001 Japan; 3 Department of Psychosomatic Research, National Institute of Mental Health, National Center of Neurology and Psychiatry, 4-1-1 Ogawahigashi, Kodaira, Tokyo, 187-8553 Japan

## Correction

After publication of the original article [1], the authors reported an error to Table 2.

**Table 2:** Spearman’s rank correlation analysis of global sleep quality (global PSQI-J)

Variables	*ρ*	*P*
Diagnosis of ANbp	0.525	0.017*
Age	0.345	0.136
Body mass index	0.168	0.479
Duration of illness	0.536	0.015*
Current menstruation	0.281	0.230
Use of sedative drugs	0.332	0.152
CES-D	0.318	0.172
Vomiting	0.561	0.010*
Chewing	-0.061	0.798
Laxative overuse	0.407	0.075
Uretic misuse	0.280	0.232

*ANbp* anorexia nervosa binge-eating/purging type; *CES-D* Center for Epidemiologic Studies Depression, *PSQI-J* Japanese version of the Pittsburgh Sleep Quality Index; *ρ* Spearman’s correlation coefficient

*P < 0.05

The title for the right-hand column of Table 2 was incorrectly included as “*ρ*” when it should have been “P”. The correct version of Table 2 is included in this Correction.
